# Supplementary material for: Identification of asymptomatic Entamoeba histolytica infection by a serological screening test: A cross-sectional study of an HIV-negative men who have sex with men cohort in Japan
Source: PLoS Negl Trop Dis. 2022 Apr 25;16(4):e0009793. doi: 10.1371/journal.pntd.0009793 (PMC9071119; doi:10.1371/journal.pntd.0009793)
Supplement: S1 Data — (DOCX) [file pntd.0009793.s001.docx]

**Supplementary data 1. Comparison of characteristics between antibody positive and negative participants.**

| Median [IQR] or % (N) | AmebaAb (-)  (N=291) | AmebaAb (+)  (N=21) | *P value* |
| --- | --- | --- | --- |
| Age | 34 [28-40] | 41 [33-47] | *< 0.01* |
| Sexual partner |  |  | *NS* |
| Male only | 91.0% (262/288) | 95.2% (20/21) |  |
| Male and female | 9.0% (26/288) | 4.8% (1/21) |  |
| Insertive/receptive |  |  | *NS* |
| Insertive only | 18.1% (52/287) | 38.1% (8/21) |  |
| Receptive only | 27.5% (79/287) | 19.0% (4/21) |  |
| Both | 51.6% (148/287) | 42.9% (9/21) |  |
| No insertive sex | 2.8% (8/287) | 0.0% (0/21) |  |
| Number of sexual partners within 6 months | 5 [3-10] | 5 [2-8] | *NS* |
| Condom use (%) | 60 [20-90] | 50 [10-80] | *NS* |
| Past treatment of any STIs | 51.5% (150/291) | 38.1% (8/21) | *NS* |
| Past treatment of amebiasis | 0.0% (0/291) | 0.0% (0/21) | *NS* |

Abbreviations: IQR, inter quartile range; N, number; STIs sexually transmitted infections; AmebaAb, anti-*Entamoeba histolytica* antibody.
