# Supplementary figures and images for: Identification of asymptomatic Entamoeba histolytica infection by a serological screening test: A cross-sectional study of an HIV-negative men who have sex with men cohort in Japan
Source: PLoS Negl Trop Dis. 2022 Apr 25;16(4):e0009793. doi: 10.1371/journal.pntd.0009793 (PMC9071119; doi:10.1371/journal.pntd.0009793)

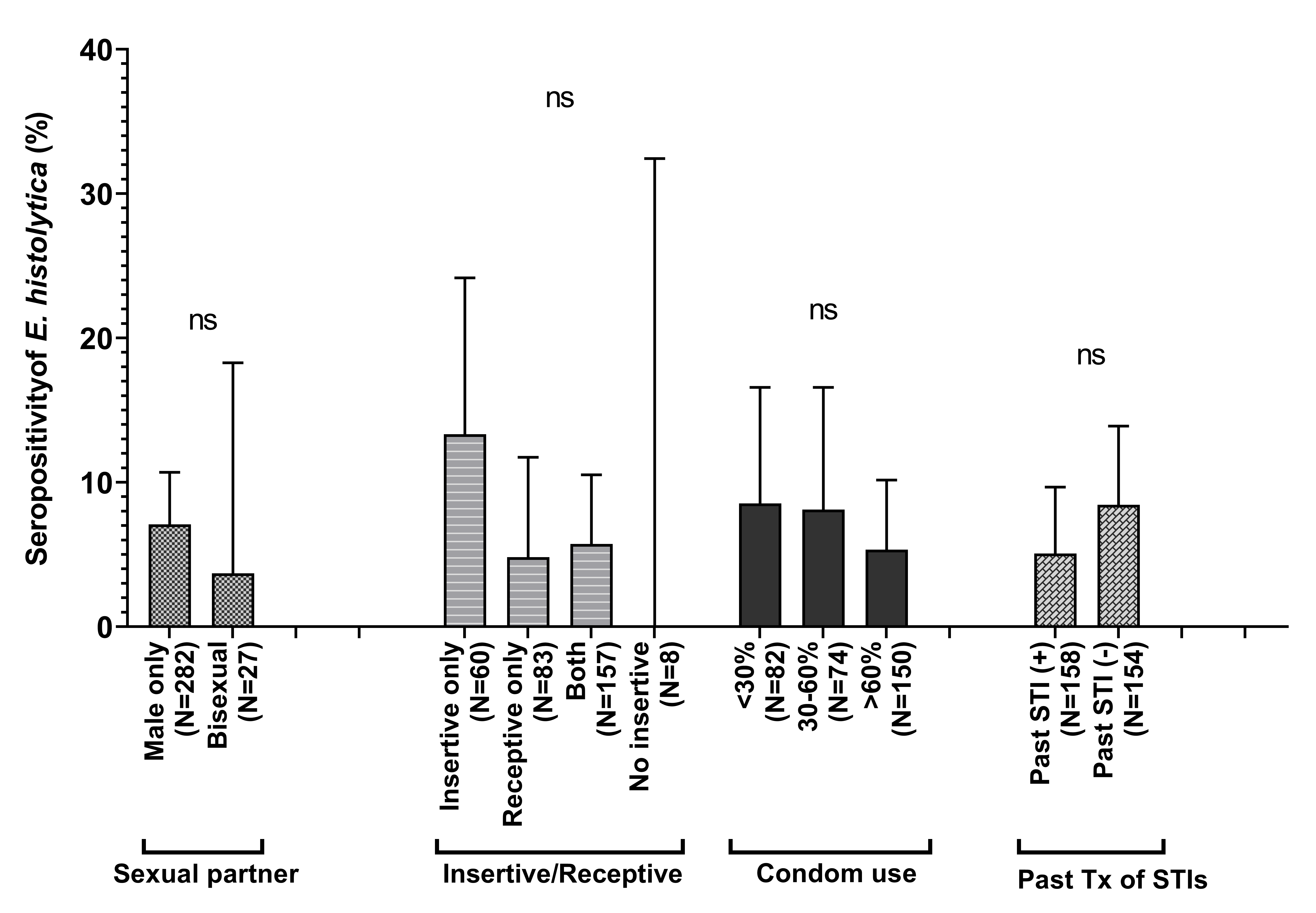

Supplement: S2 Data — There were no significant correlations between the seropositivity and sexual preferences by Fisher’s exaxt test or ANOVA test. Error bars indicate 95% confidence intervals. Abbreviations: E. histolytica, Entamoeba histolytica; STI, sexually transmitted infection; Tx, treatment history; NS, not significant. (TIF) [file pntd.0009793.s002.tif]
