# Supplementary material for: Identification of asymptomatic Entamoeba histolytica infection by a serological screening test: A cross-sectional study of an HIV-negative men who have sex with men cohort in Japan
Source: PLoS Negl Trop Dis. 2022 Apr 25;16(4):e0009793. doi: 10.1371/journal.pntd.0009793 (PMC9071119; doi:10.1371/journal.pntd.0009793)
Supplement: Attachment — Submitted filename: PNTD-D-21-01330_Point by point_Yanagawa.docx [file pntd.0009793.s004.docx]

Point-by-point responses to the comments of Reviewer #1:

We appreciate your kind suggestions for improving our manuscript. The manuscript was revised according to the comments, as shown below.

**Comments from the Reviewer #1**

1. **In Table 1, the proportions of individuals with or without antibody should be shown in each category. For example, in male only individuals as sexual partner, the proportion of antibody (+) should be calculated as 20/282 (7.1%) and that of antibody (-) should be 262/282 (92.9%). In individuals with male and female as sexual partner, the proportion of antibody (+) should be calculated as 1/27 (3.7%) and that of antibody (-) should be 26/27 (96.3%). As such, I request to recalculate the proportion of antibody (+) and (-) with making the total number of each category as a denominator.**

Response: As suggested, we modified the title and composition of Table 1. In addition, we added new Supplementary Figure 1, which compares the proportion of antibodies (+) (lines 170-172, Table 1, Supplementary Figure 1).

**Major points**

2. **Would you describe why HIV-negative men who have sex with men (MEM) can be a target for the screening of asymptomatic Entamoeba histolytica infection, in addition to the reason that “sexually active individuals”?**

Response: From the analyses of the national surveillance data, it was reported that much of the proportion of Amebiasis is MSM. We added one sentence citing two references of the national surveillance data of our country (lines 69-71, reference 5, 6).

3. **How was the cohort of HIV-negative MEM set? Can you show the outline of this cohort?**

Response: As suggested, we have modified the sentence in the Methods section to show the outline of our cohort study (lines 99-106). This cohort was a single-center prospective study. It was established to perform HIV screening and serological testing for syphilis and rectal *C. trachomatis* and *N. gonorrhoeae* every 3 months for HIV-negative MSM in 2017. Inclusion criteria of the HIV-negative cohort were MSM, aged ≥16 years old, those who have anal sexual intercourse, and who gave written informed consent for the study. People who were diagnosed with HIV at enrollment were excluded from the cohort and were referred to a HIV-positive clinic, the AIDS Clinical Center at NCGM, or other medical institutions.

4. **Do 312 participants represent all of HIV-negative MEM from the cohort? If 312 participants are a part of the cohort, how did the authors select 312 HIV-negative MEM from the cohort?**

Response: As described in the Methods section, we enrolled “all” participants who were in the HIV-negative MSM cohort between January and March 2021 (lines 97-106).

5. **In Table 1, the proportions of individuals with or without antibody should be shown in each category. For example, in male only individuals as sexual partner, the proportion of antibody (+) should be calculated as 20/282 (7.1%) and that of antibody (-) should be 262/282 (92.9%). In individuals with male and female as sexual partner, he proportion of antibody (+) should be calculated as 1/27 (3.7%) and that of antibody (-) should be 26/27 (96.3%). As such, I request to recalculate the proportion of antibody (+) and (-) with making the total number of each category as a denominator.**

Response: Thank you for this important suggestion. As reviewer 1 also commented on the same point (Question 1), we added a new Supplementary figure to the Results section (lines 170-172) and modified the title and composition of Table 1.

**Minor points**

6. **Line 102: Would you describe the name of E. histolytica antigen(s) and the concentration of the pre-coated antigen(s)?**

Response: We checked the manufacturer’s instructions again; however, detailed information regarding the *E. histolytica* antigen was not stated.

7. **Line 117: Would you describe “stool ova and parasite examination” more accurately or scientifically in detail?**

Response: As suggested, we have modified the sentence in the Methods section to describe microscopic method used in detail (lines 127-128).

8. **Line 132: Would you describe the methods and results of the DNA sequencing, if the PCR products were confirmed by sequencing?**

Response: As suggested, we have added more details to the Methods section regarding the DNA sequencing (lines 143-145).

9. **Line 143: Would you describe what kinds of STIs the 159 participants experienced?**

Response: As suggested, we have added the details of 159 past STIs to the legend of Table 1.

10. **Line 150-: Would you describe the abbreviations you described in the Fig 2B, like Treponema pallidum hemagglutination (TPHA) etc? The name of species should write down in Italic.**

Response: As suggested, we modified the name of species using italics.

11. **Line 152: Whould you clarify how the participants were “affected”?**

Response: In the paragraph, we revealed that the study participants had a high serologic prevalence of STIs including *E. histolytica* infection. However, it is still unclear whether the participants were affected because of the cross-sectional study design. We deleted the sentence regarding our speculation (line 175).

12. **Line 181-184: It is not easy to understand the meaning of the sentence.**

Response: We agree the sentence was difficult for readers to understand. We aimed to briefly summarize the results of serologic and stool PCR tests. Thus, we rewrote it to show the tests required to identify one asymptomatic *E. histolytica* infection case in the Results section (lines 211-214).

13. **Would you edit “to identify asymptomatic E. histolytica infection” --> “to identify at least an asymptomatic E. histolytica infection”**

Response: In the present cohort, all participants were checked to determine whether they had any medical problems or symptoms when visiting our hospital. We confirmed that all seropositive participants were asymptomatic at the time of *E. histolytica* testing. Therefore, the suggested edit is not appropriate.

Point-by-point response to the comments of Reviewer #2:

We appreciate your kind comments and suggestions. The manuscript has been modified according to these suggestions.

**1. This is an descriptive study for MSM people where the authours have found a seropositive for E. histolytica . 40% of the seropositive patients also positive for E. histolytica in their stool samples.**

**L-117,118 “In addition to stool ova and parasite examination (O&P)”- This line the authours has to clarify**

Response: As suggested, we have modified the sentence in the Methods section to

describe the microscopic method used in detail (lines 127-128).

**L-123 “eluted in 100 mL of elution buffer”- pleasae acheck this line**

Response: As suggested, we corrected the units to µL (line 133).

**L-131,132 “Finally, the results were confirmed by DNA sequencing”- should include**

**briefly the procedures or a reference of the method.**

Response: As suggested, we have added more detail regarding the DNA sequencing in the Methods section (lines 143-145).

2. **L-143 “none had a history of E. histolytica infection”- How did the author confirm this? There is no precise statement**

Response: In the present study, we checked the “treatment” histories of all participants using a medical self-declaration form. As stated by the reviewer, we could not confirm that they had never been infected with *E. histolytica* because most cases are asymptomatic. We modified the sentence to clarify this issue (line 164-165).

**L-147 “hepatitis B surface antigen and hepatitis C virus antibody”- Which methods were utilized to find those? Should include in methods section.**

Response: As suggested, we have added more detail regarding STI testing in the Methods section (lines 146-153).

**L-175,176 “One of the 21 seropositive individuals refused stool examination”- therefore, author examined a total of 20 stool samples. In this case, PCR detected 40% E. histolytica, yet the author computed all 21 samples in table 1 –should it be revised**

Response: As you correctly pointed out, we should have analyzed 20 stool samples in Table 1. Thus, we simplified the composition of Table 1.

3. **The manuscript does not provide any justification how E. histolytica infections correlate with STI’s. Since this study focused on active epidemiological surveys, the author should specify the number of people that tested positive for rapid plasma reagin (RPR) out of the 21 Eh seropositive samples.**

Response: As described in the Results section and Figure 2A, the percentage of people that tested positive for rapid plasma reagin (RPR) out of the 21 Eh seropositive samples was 5.4% (17/312). This seroprevalence was similar to that of *E. histolytica* in this study. We added the RPR positive percentage to the Results section (line 167).
