# Supplementary material for: Identification of asymptomatic Entamoeba histolytica infection by a serological screening test: A cross-sectional study of an HIV-negative men who have sex with men cohort in Japan
Source: PLoS Negl Trop Dis. 2022 Apr 25;16(4):e0009793. doi: 10.1371/journal.pntd.0009793 (PMC9071119; doi:10.1371/journal.pntd.0009793)
Supplement: Attachment — Submitted filename: R2_PNTD-D-21-01330_4_Point by point responses.docx [file pntd.0009793.s005.docx]

Point-by-point responses to the comments of Reviewer #3:

We appreciate your kind suggestions for improving our manuscript. The manuscript was revised according to the comments, as shown below.

**Comments from the Reviewer #3**

1. **The major weakness of this study was that only the participants who were seropositive for E. histolytica underwent PCR assay to identify intestinal infection with E. histolytica. Given the high sensitivity and specificity of PCR assay for detection of E. histolytica, PCR assay should be considered as gold standard for the diagnosis of intestinal infection with E. histolytica. All recruited subjects should undergo PCR assay of stool samples, followed by serologic assay to better understand the performance of serologic assay used in this study. With the understanding of the performance of serologic assay could the authors be able to examine the “effectiveness” of serologic assay in identifying high-risk individuals with E. histolytica infection. It is understandable that serologic screening would be cheaper and more simple and convenient to perform than PCR assay of stool samples from clinicians’ standpoint; however, to better examine the “effectiveness” or “cost-effectiveness” of serologic testing that is to be widely used for screening in the clinical settings, both PCR assay of stool samples and serologic testing of all recruited participants, not selected participants, should be performed side by side. While the authors discussed it as a limitation, the last several sentences (Lines 218-221) in Results and Conclusions could be incorrect without testing all participants with the use of PCR assay.**

Response: Thank you for these comments. We completely agree that our study design was insufficient to calculate the sensitivity and specificity of serological screening for the detection of *E. histolytica*. However, we think the present study still has value because this is the first study to report that prospective serological screening successfully identified *E. histolytica* infected cases in a high-risk but asymptomatic population. In many developed countries, especially in East Asia and Australia, the high seroprevalence of *E. histolytica* has been reported among MSM and CSW (female commercial sex workers). However, most seropositive individuals are asymptomatic and have no history of amebiasis. Additionally, seropositivity of *E. histolytica* cannot differentiate current infection from past infection. However, our recent data showed that serum antibody tests are highly sensitive for invasive disease and asymptomatic infection by *E. histolytica*, although it is still unknown whether serological testing can be a screening method for asymptomatic infection (sentences in the Background section were modified [lines 86–89]). From the view point of above, this is the first study showing that serological screening can identify asymptomatic *E. histolytica* infected cases from a population at high risk for sexually transmitted *E. histolytica* infection (MSM). As you commented, the effectiveness of antibody screening tests cannot be assessed unless the performance of the PCR assay and serologic assay is determined for all participants. Therefore, we modified the title, the descriptions in the Results (lines 223–226), and those in the Discussion (lines 237–239, 243–246).

2. **The authors are encouraged to provide the sample size estimation. It is not clear that how many MSM had been recruited for STI studies at the voluntary counseling and testing site and how many agreed to participate in serologic and PCR testing in this cross-sectional survey.**

Response: As suggested, we added the sample size estimation to the Methods section (lines 109–120).

3. **While testing for gonorrhea, syphilis and chlamydia was performed every 3 months, it sounds that serologic testing for E. histolytica was only performed at baseline. Did the authors follow the participants using the same serologic assay to estimate the seroconversion rate?**

Response: As described in the Methods section, this was a cross-sectional study using a pre-existing MSM cohort (lines 97–99). We performed serological testing for *E. histolytica* only once during the study period. Calculation of the seroconversion rate of *E. histolytica* is not available and is out of the scope of the present study.

4. **In Table 1, 3 out of 311 participants had had treatment of amebiasis. In the footnote, 4 individuals had had amebiasis as past sexually transmitted infections (STIs). The authors are encouraged to provide more information on these participants who either had had treatment or amebiasis as STIs because none were reported to have had previous E. histolytica infection in Results section. Moreover, the statement (Lines 227-228) in the first paragraph of Discussion is contradictory to the data presented in Table 1.**

Response: As pointed out, the number of participants with a history of amebiasis was wrongly filled in at the first revision (R1). None of the participants had a history of amebiasis at the serological screening test. We corrected the numbers in Table 1. Additionally, the footnote of Table 1 was corrected (lines 208–212, Supplementary data 3).

5. **Were the ELISA units of the participants testing positive for E. histolytica by PCR assay higher than those of participants testing seropositive but negative by PCR assay?**

Response: Although data were not presented in the manuscript, there was no statistically significant difference between PCR-positive and -negative participants (18.25 in the PCR positive group vs 16.92 in the PCR negative group, p 0.675).

6. **Table 1 could be improved by providing data of “all participants”, “E. histolytica-seropositive participants”, and “E. histolytica-seronegative participants”, with p-values for the comparisons between the latter two groups.**

Response: At the first revision, we modified the contents of Table 1 from a comparison of characteristics between seropositive and seronegative individuals to the current format according to a suggestion by Reviewer #1. We agree with the current format in Table 1 that shows the basic characteristics of study participants. Instead of modifying Table 1, we have added Supplementary data 1, which compares the characteristics between seropositive and seronegative participants.

7. **In Table 1, should “number of sex within 6 months” be “number of sexual partners within 6 months”?**

Response: Thank you for this comment. As suggested, the description in Table 1 was corrected.

8. **While the authors discussed it as a limitation, the last several sentences (Lines 218-221) in Results and Conclusions could be incorrect without testing all participants with the use of PCR assay.**

Response: As suggested, we modified the title, descriptions in the Results (lines 223–226), and those in the Discussion (lines 237–239, 243–246). Please also see our responses to “comment 1” for more details.

9. **The authors performed a cross-sectional serologic survey of E. histolytica infection among 312 HIV-negative men who have sex with men (MSM) and had had no known previous history of E. histolytica infection in Tokyo. Polymerase-chain-reaction assay was performed only in those 20 individuals who tested seropositive for E. histolytica, in which 8 (40%) tested positive for E. histolytica, suggesting current infection. While the data may be interesting from the perspectives of E. histolytica infection remaining prevalent among MSM in developed countries, particularly in East Asia, there are major concerns regarding the study design.**

Response: Thank you for the kind comments on the manuscript. We hope the revised manuscript is now suitable for publication.
