# Supplementary material for: Identification of asymptomatic Entamoeba histolytica infection by a serological screening test: A cross-sectional study of an HIV-negative men who have sex with men cohort in Japan
Source: PLoS Negl Trop Dis. 2022 Apr 25;16(4):e0009793. doi: 10.1371/journal.pntd.0009793 (PMC9071119; doi:10.1371/journal.pntd.0009793)
Supplement: Attachment — Submitted filename: R3_PNTD-D-21-01330_4_Point by point responses.docx [file pntd.0009793.s006.docx]

Point-by-point responses to the comments of Reviewer #2:

We appreciate your kind suggestions for improving our manuscript. The manuscript was revised according to the comments, as shown below.

**Comments from the Reviewer #2**

1. **Authors can take out the last sentence of the Results section and use it in the discussion section. The last sentence of the abstract section is not required authors may take it out. This sentence is also included in the result section but, authors can take it to the Discussion section.**

Response: As suggested, we have deleted the last sentence of the Methodology/Principal Findings in Abstract section and took it to the Discussion section (lines 231–234).
